# A Novel STAT3-Mediated GATA6 Pathway Contributes to *tert*-Butylhydroquinone- (tBHQ-) Protected TNF*α*-Activated Vascular Cell Adhesion Molecule 1 (VCAM-1) in Vascular Endothelium

**DOI:** 10.1155/2020/6584059

**Published:** 2020-11-14

**Authors:** Li Zhou, Hua Ning, Haibin Wei, Tiantian Xu, Xindi Zhao, Ai Fu, Qianyu Qian, Zhen Yang, Xiaobing Dou, Songtao Li

**Affiliations:** ^1^College of Basic Medicine & Public Health, Zhejiang Chinese Medical University, Hangzhou 310053, China; ^2^College of Life Science, Zhejiang Chinese Medical University, Hangzhou, Zhejiang 310053, China; ^3^The First Affiliated Hospital of Zhejiang Chinese Medical University, Hangzhou 310000, China; ^4^Molecular Medicine Institute, Zhejiang Chinese Medical University, Hangzhou, Zhejiang 310053, China

## Abstract

The activation of vascular cell adhesion molecule 1 (VCAM-1) in vascular endothelial cells has been well considered implicating in the initiation and processing of atherosclerosis. Oxidative stress is mechanistically involved in proatherosclerotic cytokine-induced VCAM-1 activation. *tert*-Butylhydroquinone (tBHQ), a synthetic phenolic antioxidant used for preventing lipid peroxidation of food, possesses strongly antioxidant capacity against oxidative stress-induced dysfunction in various pathological process. Here, we investigated the protective role of tBHQ on tumor necrosis factor alpha- (TNF*α-*) induced VCAM-1 activation in both aortic endothelium of mice and cultured human vascular endothelial cells and uncovered its potential mechanisms. Our data showed that tBHQ treatment significantly reversed TNF*α*-induced activation of VCAM-1 at both transcriptional and protein levels. The mechanistic study revealed that inhibiting neither nuclear factor (erythroid-derived 2)-like 2 (Nrf2) nor autophagy blocked the beneficial role of tBHQ. Alternatively, tBHQ intervention markedly alleviated TNF*α*-increased GATA-binding protein 6 (GATA6) mRNA and protein expressions and its translocation into nucleus. Further investigation indicated that tBHQ-inhibited signal transducer and activator of transcription 3 (STAT3) but not mitogen-activated protein kinase (MAPK) pathway contributed to its protective role against VCAM-1 activation via regulating GATA6. Collectively, our data demonstrated that tBHQ prevented TNF*α*-activated VCAM-1 via a novel STAT3/GATA6-involved pathway. tBHQ could be a potential candidate for the prevention of proatherosclerotic cytokine-caused inflammatory response and further dysfunctions in vascular endothelium.

## 1. Introduction

Cardiovascular diseases (CVDs) rank the leading cause of human mortality in both developed and developing countries [1]. Atherosclerosis (AS) per se is not only a serious cardiovascular disease but also an important incentive of other cardiovascular events, including coronary heart disease, cerebral infarction, and peripheral vascular disease [2]. The activation of adhesion molecules in vascular endothelial cells has been implicated as the early step in the pathological process of AS [3, 4]. Several crucial adhesion molecules have been identified in the past decades, including vascular cell adhesion molecule 1 (VCAM-1), intercellular adhesion molecule 1 (ICAM-1), and E-selectin; among which, VCAM-1 has been well recognized as playing a significant role in the initiation of atherosclerosis [4–6], where its induction further recruits blood monocyte adhesion to the endothelial cells via interaction with the integrin *α*4*β*1 and transmigration across the vascular endothelium [7]. VCAM-1 mutation significantly alleviated cholesterol-enriched diet-induced early atherosclerotic lesions in low-density lipoprotein (LDL) receptor null mouse [5]. Antagonizing VCAM-1 by its special monoclonal antibody obviously reduced high-cholesterol diet-enhanced monocyte/macrophage infiltration and neointimal formation in apolipoprotein E null mice [8]. The above evidences strongly suggested that VCAM-1 could be an effective therapeutic target for AS.

VCAM-1 is mainly secreted from vascular endothelial cells. In normal physiological state, vascular endothelial cells are in a resting state, and VCAM-1 is scarcely detected in cultured vascular endothelial cells. However, VCAM-1 could be instantly transcriptionally stimulated by proatherosclerotic cytokines, such as tumor necrosis factor-alpha (TNF*α*), IL-1-beta (IL-1*β*), angiotensin, and lipopolysaccharide (LPS), in both human and experimental animal models [9–14]. It has been well documented that VCAM-1 could be transcriptionally activated by several nuclear transcription factors, including nuclear factor kappa-B (NF*κ*B), interferon regulatory factor-1 (IRF-1), GATA-binding proteins (GATAs), specificity protein-1 (SP-1), and activator protein-1 (AP-1, including c-Fos and c-Jun) in different pathological processes [15–19]. Genetically or chemically inhibiting nuclear transcription factors mentioned above dramatically blocked VCAM-1 activation caused by various stimuli [20–29].

*tert*-Butylhydroquinone (tBHQ), a synthetic phenolic antioxidant, is widely used as food additive to prolong food preservation. The application of tBHQ in human beings has been proved safe and approved by the Food and Agriculture Organization (FAO) and World Health Organization (WHO). Accumulated evidences have indicated that tBHQ not only preserves food ingredients from oxidation but also protects oxidative stress-induced dysfunction in mammalian cells and tissues [30–32]. It is well accepted that tBHQ exerts its antioxidative function through enhancing nuclear factor (erythroid-derived 2)-like 2 (Nrf2) protein stability via repressing of the Kelch-like ECH-associated protein 1- (Keap1-) mediated ubiquitination [33]. In resting state, Keap1 functions as an anchor binding with Nrf2 and promotes Nrf2 degradation by ubiquitination degradation pathway [34]. After dissociation from Keap1, Nrf2 translocates into nucleus and exerts its antioxidative role via activating phase II detoxification enzymes and certain antioxidants through binding with the antioxidant response element (ARE) in the promotor sequence [35, 36]. Previous studies revealed that Nrf2 activation is an effective strategy for the prevention of vascular endothelial cell dysfunction and further progress of AS [36–40]. Nrf2 has been proven to protect VCAM-1 activation in TNF*α*-exposed human aortic endothelial cells (HAEC) and AS model rats [41, 42]. Besides, tBHQ has exhibited some cardiovascular protective effects, including improving ischemia-induced angiogenesis and heart functions in hypertensive rats and attenuating oxidative stress and inflammation in hypothalamic paraventricular nucleus in high salt-induced hypertension [43, 44]. However, little is known about the protective effect of tBHQ against VCAM-1 activation in vascular endothelial cells.

We previously reported that tBHQ is a strong inducer of autophagy in hepatocytes [45]. Recent works indicated that activating autophagy prevented TNF*α*-induced VCAM-1 activation in vascular endothelial cells [21, 46]. We also observed that tBHQ-induced Nrf2 activation depends on autophagy induction [45]. Therefore, we presumed that tBHQ protects VCAM-1 activation via inducing autophagy- and/or Nrf2-dependent pathway.

As expected in the present study, we observed that tBHQ treatment dramatically inhibited TNF*α*-induced VCAM-1 activation in both aortic endothelium of mice and cultured human vascular endothelial cells. Unexpectedly, neither Nrf2 nor autophagy activation contributed to the protective role of tBHQ. Our mechanistic study provided a novel STAT3/GATA6 pathway conferred to the beneficial effect of tBHQ against TNF*α*-induced VCAM-1 activation. Our study obtained another layer of mechanism to the established beneficial effects of tBHQ and expanded the cognition of biological function of tBHQ in improving endothelial dysfunction.

## 2. Material and Methods

### 2.1. Reagents

tBHQ, rapamycin, and hydroxychloroquine sulfate (CQ) were obtained from Sigma Aldrich (Sigma Aldrich, MO). TRIzol™ Reagent, RNAiMAX, and Rapid Gold BCA Protein Assay Kit were provided by Thermo Fisher (Thermo Fisher Scientific, MA). STAT3 inhibitor Stattic, SHP2 inhibitor SHP099, p38 inhibitor SB202190, and ERK1/2 inhibitor FR180204 were bought from Selleck (Selleck Chemicals, TX). Recombined TNF*α* (both human and mouse) was bought from R&D Systems (R&D Systems, MN). tBHQ was dissolved in ethanol; hydroxychloroquine sulfate was dissolved in sterilized water, and other compounds were dissolved in dimethyl sulfoxide (DMSO). VCAM-1 antibody, c-Fos antibody, and p38 activator U46619 were purchased from Abcam (Cambridge, UK); histone H3 antibody was obtained from Protein-tech (Protein-tech, IL). GATA6, ATG7, phospho-p38, total-p38, phospho-Y705-STAT3, total-STAT3, NF*κ*B (p65), SP-1, c-Jun, Nrf2, phospho-SHP2, and total SHP2 antibodies were purchased from Cell Signaling Technology, Inc. (Cell Signaling Technology, MA); LC3 and peroxidase conjugated *β*-actin antibodies were obtained from Sigma Aldrich (Sigma Aldrich, MO); horseradish peroxidase conjugated secondary antibodies was purchased from Jackson ImmunoResearch Inc. (Jackson ImmunoResearch, PA).

### 2.2. Animals

All experimental protocols were approved by the Zhejiang Chinese Medical University Animal Care and Use Committee (ZSLL-2018-046). Twenty male C57BL/6J mice (8 weeks old) obtained from SLAC laboratory animal Co. Ltd. (Shanghai, China) were randomly divided into four groups (*n* = 5 each), including the normal control, TNF*α* treatment, tBHQ treatment, and tBHQ+TNF*α* treatment groups. tBHQ were diluted in corn oil with a final concentration of 6.25 mg·mL^−1^. TNF*α* was dissolved in 0.9% sodium chloride solution with a final concentration of 5 *μ*g·mL^−1^. Each reagent was diluted to a final volume of 0.1 mL per mouse and administered via intraperitoneal injection. tBHQ was injected with a concentration of 25 mg·kg^−1^ body weight 2 h before recombinant mouse TNF*α* (30 *μ*g·kg^−1^ body weight) injection. Each group received the same amount of solvent injection. Six hours after TNF*α* intervention, mice were anesthetized and sacrificed. Plasma and portions of aorta were harvested for further analysis. Plasma VCAM-1 was tested using a commercial ELISA kit (Cusabio Biotech, Wuhan, China) according to the manufacturer's protocol (*n* = 5).

### 2.3. Immunohistochemistry

Aortic cross-sections were fixed with paraformaldehyde for 12 h and processed for immunostaining for VCAM-1, p-STAT3, and GATA6, followed by incubation with goat anti-rabbit IgG (CWBIO, Beijing, China). After washing with PBS, color was developed with diaminobenzidine (DAB) solution (CWBIO, Beijing, China), and the nuclei were counterstained with Harris hematoxylin for 15 s. Unless specified, the sections were blocked with 1% BSA plus normal horse serum or 1% BSA alone. Negative controls were performed by omitting primary antibody, and the representative pictures were shown in Supplementary data (Figure S9). Photographs of immunostained mouse aorta (200x magnification) were digitized and captured using an inverted bright field and phase contrast microscope (Nikon Ti-S, Japan).

### 2.4. Cell Culture

Human aortic endothelial cells (HAEC) (kindly granted from Dr. Haoyuan Deng, Dalian Medical University) were cultured in M199 medium supplemented with 8% fetal bovine serum (FBS), 100 g·mL^−1^ heparin, 10 ng·mL^−1^ endothelial growth factor, 100 g·mL^−1^ hypothalamus extract, 2 mmol·L^−1^ L-glutamine, and 1% nonessential amino acids. EA.hy926 cell line was obtained from American Type Culture Collection (ATCC, Manassas, VA) and cultured in high-glucose Dulbecco's Modified Eagle Medium (DMEM, HyClone, IL) supplemented with 10% FBS (Cellmax, CA). All cells were maintained in a humidified 95% air and 5% CO_2_ atmosphere at 37°C. Cells were seeded into 100 mm tissue culture dish. After 80–90% confluence, cells were treated with tBHQ, recombinant human TNF*α*, and/or other reagents for the indicated duration. After the treatment, cells were collected for further measurements. Each *in vitro* test was performed at least 3 times.

### 2.5. Cell Viability Assay

Cell viability was verified with Cell Counting Kit-8 (CCK-8) (Bimake, TX) according to the manufacturer's instructions. In brief, 2 × 10^3^ cells per well were seeded onto 96-well plate. After all treatments, culture medium was removed and immediately added 100 *μ*L CCK-8 working solution (1 : 10 diluted with base medium, *v*/*v*) incubating at 37°C for 2 h. Absorbance data were detected at 490 nm using Synergy™H1 (BioTek, VT).

### 2.6. mRNA Analysis

Total RNA was extracted with TRIzol™ Reagent (Thermo Fisher Scientific, MA), and then, 4 *μ*g of total RNA were reverse transcribed into cDNA with All-in-One cDNA Synthesis SuperMix (Bimake, TX). SYBR green (Bimake, TX) was employed to detect the CT value with 2^−ΔΔCT^ method calculating relative gene expression level using QuantStudio7 (Thermo fisher Scientific, MA). *ACTB* was used as housekeeping gene for calibration. Primers' sequences were listed in Table 1.

### 2.7. RNA Interfering

Small interfering RNA (siRNA) for Nrf2 and GATA6 were designed and synthesized by GenePharma (Shanghai, China). RNAiMAX was utilized to deliver siRNA to the targeted cells according to the manufacturer's protocol. Scrambled siRNA (GenePharma, Shanghai, China) was applied in negative control group. The concentration of siRNAs used in this study is 10 nmol·L^−1^.

### 2.8. Western Blot Assay

Total or nuclear proteins in cultured cells were separately extracted using RIPA lysis buffer or nuclear and cytoplasmic protein extraction kit (CWBIO, Beijing, China), according to manufacturer's instructions. Detailed protocol of Western blot in our laboratory was described previously [47], blots were presented by X-ray film development (Carestream, NY). *β*-Actin or histone H3 was separately used as internal control for total or nuclear protein. Quantitative analysis of protein expression in Western blots was evaluated by Image J (NIH Image, Bethesda, MD). Each test was conducted at least 3 times, and a representative blot was shown.

### 2.9. Analysis of Autophagic Flux

The autophagic flux was measured as previously described [48]. In brief, CQ was added prior to the indicated treatment to inhibit lysosome acidification. The autophagic flux was determined by detecting GFP-LC3 puncta with laser scanning confocal microscope (LSM 880, ZEISS, Germany) as well as LC3-II, and ATG7 expression by Western blot, respectively. For GFP-LC3 fluorescence detection, cells were transiently infected with recombinant lentivirus-expressing GFP-LC3 (GeneChem, Shanghai, China). At least 50 cells were counted in each individual experiment. Mean value of puncta per cell in each group was shown in the quantification data.

### 2.10. Statistical Analysis

All data were expressed as mean ± SD. The statistical analysis was performed with the GraphPad Prism 8.02 software (GraphPad Software, CA) using *t*-test or one-way analysis of variance (ANOVA) followed by Tukey's multiple comparisons test. All *P* values were two-tailed; differences between each group were considered to be significant while *P* < 0.05.

## 3. Results

### 3.1. tBHQ Protects TNF*α*-Induced VCAM-1 in Vascular Endothelium

**Figure 1:**
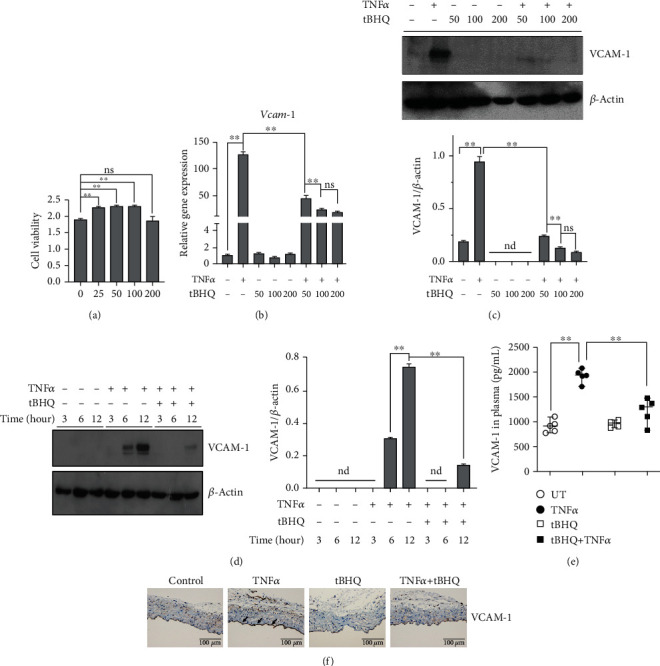
tBHQ prevents TNF*α*-induced VCAM-1 activation in vascular endothelium. HAEC cells were treated with TNF*α* (10 ng·mL^−1^, 6 h for mRNA and 16 h for protein detection). tBHQ (100 *μ*mol·L^−1^ or as indicated doses in the picture) was added 1 h before TNF*α* treatment. (a) After 24 h tBHQ treatment, cell viability of HAEC cells was detected by CCK-8 method. (b) The mRNA level of *Vcam-1*. (c) Protein expression of VCAM-1. (d) VCAM-1 expression was detected by Western blotting. (e) Plasma VCAM-1 was measured (*n* = 5). (f) Immunohistochemisty for vascular endothelium VCAM-1 expression (*n* = 5). In vitro values are denoted as means ± SD from three or more independent batches of cells. Each group contains the same amount of solvent. nd: none-detective. ^∗∗^*P* < 0.01 indicates statistically significant differences. ns: no significant differences.

The cytotoxicity of tBHQ on HAEC was first evaluated by CCK-8 test. No cytotoxicity was observed when the dose of tBHQ was less than 200 *μ*mol·L^−1^(Figure 1(a)). To test whether tBHQ confers protection against TNF*α*-induced VCAM-1 activation in HAEC, we pretreated HAEC with an incremental dose of tBHQ (0, 50, 100, and 200 *μ*mol·L^−1^) for 1 hour before 12 h TNF*α* (10 ng·mL^−1^) exposure. VCAM-1 expression was detected at both transcriptional and protein levels. Our data clearly showed that TNF*α* treatment significantly stimulated VCAM-1 activation, whereas tBHQ robustly prevented TNF*α*-induced activation of VCAM-1, with optimal dose over 100 *μ*mol·L^−1^ tBHQ (Figures 1(b) and 1(c)). We also analyzed time-course protective role of tBHQ and observed that tBHQ dramatically inhibited TNF*α*-induced VCAM-1 expression at the tested time-point (Figure 1(d)). To test the generality of our finding, we conducted the similar experiments in EA.hy926 cells (human vein endothelial cell line). Our results showed that TNF*α*-stimulated VCAM-1 activation in human vein endothelial cells was also significantly inhibited by tBHQ intervention (Figure S2). More importantly, TNF*α* injection-induced increase of VCAM-1 in both plasma and aortic endothelium was strongly reversed by tBHQ intervention (Figures 1(e) and 1(f)).

### 3.2. The Protective Role of tBHQ Is Independent from Nrf2 Activation

**Figure 2:**
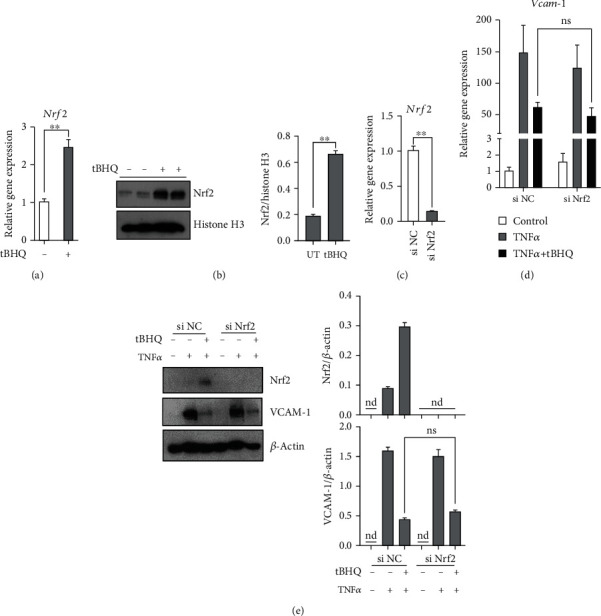
Nrf2 activation is not involved in tBHQ-exerted protection against VCAM-1 activation. HAEC cells were transfected with siNrf2 or scramble siRNA (siNC) and followed by TNF*α* (10 ng·mL^−1^, 6 h for mRNA and 16 h for protein detection) treatment. tBHQ (100 *μ*mol·L^−1^) was added 1 h before TNF*α* treatment. (a) The mRNA level of *Nrf2* was detected after 24 h tBHQ treatment. (b) Nrf2 protein expression was detected after 24 h tBHQ treatment. (c) Silence efficiency was evaluated by detecting mRNA level of *Nrf2*. (d) The mRNA level of *Vcam-1* was detected. (e) VCAM-1 expression was detected. All values are denoted as means ± SD from three or more independent batches of cells. Each group contains the same amount of solvent. nd: none-detective. ^∗^*P* < 0.05 and ^∗∗^*P* < 0.01 indicate statistically significant differences. ns: no significant differences.

tBHQ has been well considered as a Nrf2 inducer. In line with previous studies, data from our experiments indicated that tBHQ treatment significantly increased Nrf2 mRNA expression and protein abundance in nucleus (Figures 2(a) and 2(b)). To clarify whether Nrf2 activation was involved in the protective role of tBHQ, Nrf2-specific siRNA was transfected into HAEC for Nrf2 gene silencing. The transcriptional level of *Nrf2* was significantly decreased after siRNA transfection (Figure 2(c)); however, the siRNA-mediated Nrf2 gene silencing did not block tBHQ-protected VCAM-1 activation induced by TNF*α*, which indicated that tBHQ protected HAEC against VCAM-1 activation via a Nrf2-indepentdent mechanism (Figures 2(d) and 2(e)).

### 3.3. Autophagy Activation Is Not Involved in the Protective Effect of tBHQ

**Figure 3:**
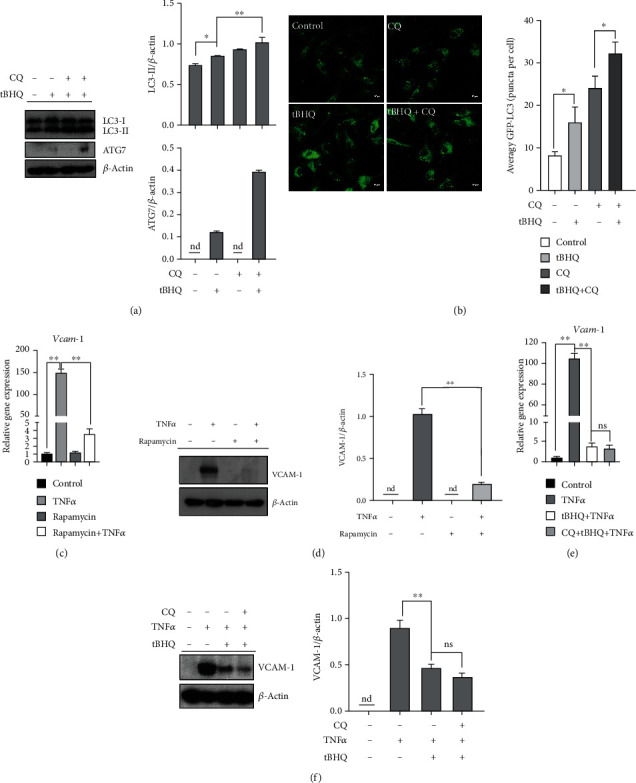
Autophagy induction is negatively associated with tBHQ-prevented VCAM-1 activation. HAEC cells were treated with TNF*α* (10 ng·mL^−1^, 6 h for mRNA and 16 h for protein detection). tBHQ (100 *μ*mol·L^−1^) was added 1 h before TNF*α* treatment. Rapamycin (200 nmol·L^−1^) was added 1 h before TNF*α* treatment. CQ (20 *μ*mol·L^−1^) was added 1 h before tBHQ intervention. (a) LC3 and ATG7 expressions were detected by Western blotting after 16 h tBHQ treatment. (b) LC3 puncta were detected by confocal microscope after 16 h tBHQ treatment. (c) mRNA of *Vcam-1*. (d) VCAM-1 expression was detected by Western blotting. (e) mRNA of *Vcam-1*. (f) VCAM-1 expression was detected by Western blotting. All values are denoted as means ± SD from three or more independent batches of cells. Each group contains the same amount of solvent. nd: none-detective. ^∗^*P* < 0.05 and ^∗∗^*P* < 0.01 indicate statistically significant differences. ns: no significant differences.

We previously reported that tBHQ is a strong autophagy inducer in hepatocytes [45]. Comprehensively considering that activating autophagy can improve VCAM-1 activation induced by TNF*α* [21, 46], the possibility of tBHQ on improving TNF*α*-induced VCAM-1 via activating autophagy was further analyzed. tBHQ treatment significantly increased protein abundance of autophagosome-associated microtubule-associated protein 1 (MAP1) light chain 3 (LC3)-II and ATG7 in HAEC (Figure 3(a)). Autophagic flux was also activated by tBHQ exposure, evidenced by the observation of more LC3-II abundance, as well as LC3 puncta in the presence of lysosomal acidification blocker, CQ (Figure 3(b)). Besides, rapamycin, a specific chemical agonist of autophagy, markedly inhibited TNF*α*-induced VCAM-1 activation at both transcriptional and protein levels (Figures 3(c) and 3(d)). However, CQ, specific inhibitors of autophagy, failed to block the protective role conferred by tBHQ (Figures 3(e) and 3(f)). To consolidate our observations, we also tested the effect of autophagy on tBHQ protected VCAM-1 in EA.hy926 cells. Similarly, inhibiting autophagy did not affect the protection of tBHQ against TNF*α*-induced VCAM-1 (Figure S3), which indicated that autophagy activation was not involved in tBHQ-protected VCAM-1.

### 3.4. The Reduction of GATA6 Translocation into Nucleus Contributes to the Protection of tBHQ

**Figure 4:**
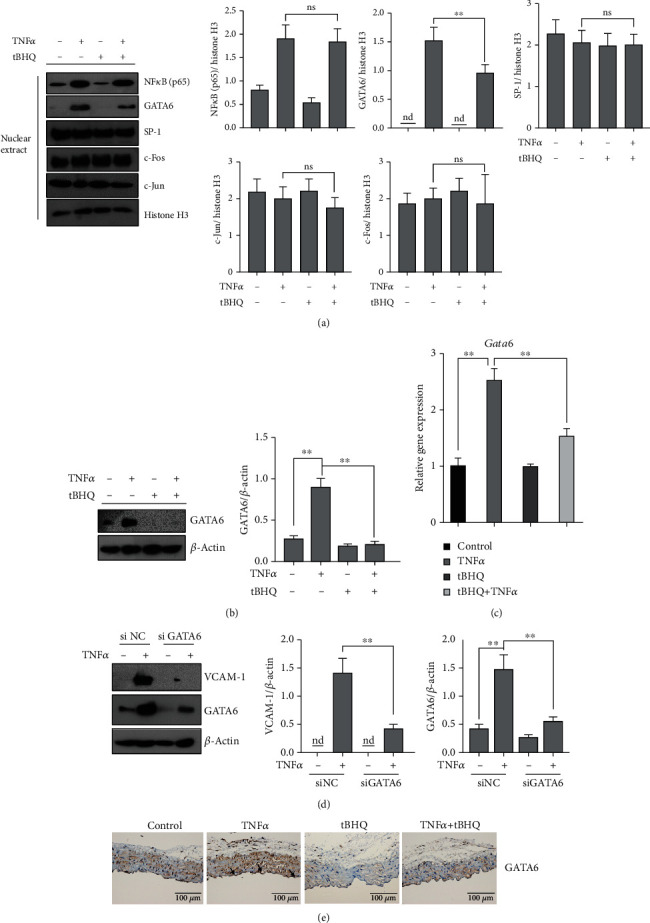
tBHQ inhibits TNF*α*-activated GATA6 in vascular endothelial cells. HAEC cells were treated with TNF*α* (10 ng·mL^−1^, 6 h for mRNA and 16 h for protein detection). tBHQ (100 *μ*mol·L^−1^) was added 1 h before TNF*α* treatment. (a) Nuclear protein was exacted after the indicated treatment. Western blotting was performed to detect the expression of NF*κ*B (p65), GATA6, SP-1, c-Fos, and c-Jun in nucleus. (b) Total GATA6 expression. (c) mRNA level of *Gata*6 was detected. (d) HAEC cells were transfected with si GATA6 or scramble siRNA (si NC) and followed by TNF*α* treatment for 16 h. Western blotting was performed to detect the intracellular expression of VCAM-1 and GATA6. (e) Immunohistochemisty for vascular endothelium GATA6 expression (*n* = 5). In vitro values are denoted as means ± SD from three or more independent batches of cells. Each group contains the same amount of solvent. nd: none-detective. ^∗∗^*P* < 0.01 indicates statistically significant differences. ns: no significant differences.

To further analyze the potential mechanisms through which tBHQ protected against TNF*α*-induced VCAM-1 activation in vascular endothelium, the well-recognized nuclear transcriptional factors for VCAM-1, including NF*κ*B (p65), GATA6, SP-1, c-Fos, and c-Jun, were screened subsequently. TNF*α* exposure markedly increased intranuclear metastasis of NF*κ*B (p65), and GATA6, but not SP-1, c-Fos, and c-Jun in HAEC (Figure 4 a), whereas only GATA6 was reduced by tBHQ pretreatment (Figure 4(a)). Similar results were also observed in EA.hy926 cells (Figure S4). Interestingly, we found that TNF*α* exposure not only prompted GATA6 entering into the nucleus but also increased its intracellular protein abundance (Figure 4(b)). Further, we observed that *Gata6* could be transcriptionally regulated by TNF*α* intervention (Figure 4(c)). Silencing GATA6 by its special siRNA obviously blocked TNF*α*-induced activation of VCAM-1 (Figure 4(d)). Besides, tBHQ treatment robustly reversed TNF*α*-increased intracellular content of GATA6 at both transcriptional and protein levels (Figures 4(b) and 4(c)). More importantly, tBHQ intervention robustly reversed TNF*α* injection-induced increase of GATA6 expression in aortic endothelium (Figure 4(e)), which indicated that the downregulation of GATA6 by tBHQ contributed to its protective role.

### 3.5. p38 MAPK Is Independently Associated with tBHQ-Inhibited GATA6 Expression

**Figure 5:**
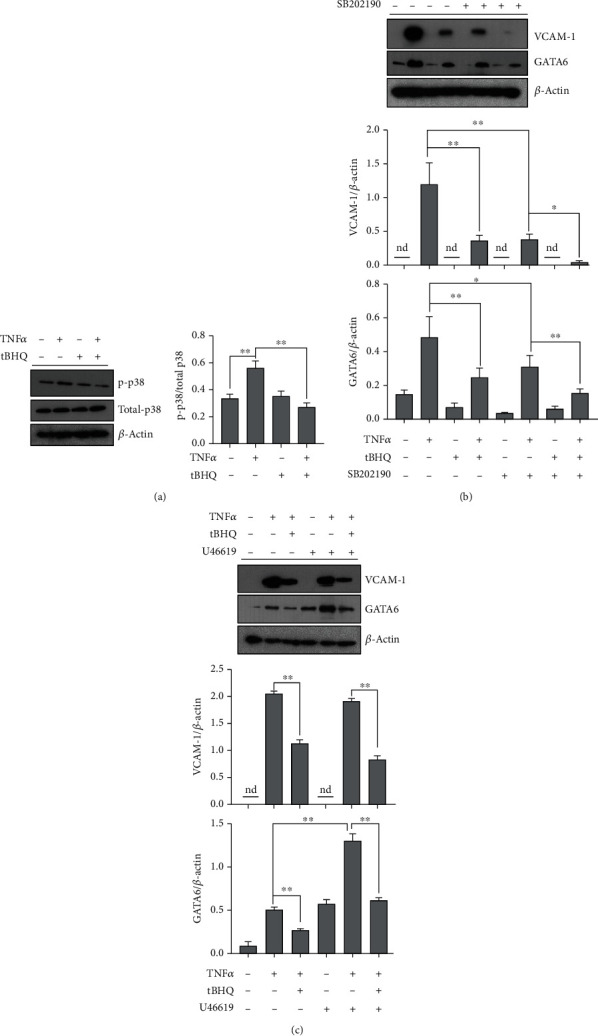
MAPK pathway is negatively associated with tBHQ-decreased GATA6. HAEC cells were treated with TNF*α* (10 ng·mL^−1^) for 16 h. tBHQ (100 *μ*mol·L^−1^) was added 1 h before TNF*α* treatment. SB202190 (5 *μ*mol·L^−1^) or U46619 (10 nmol·L^−1^) was added 1 h before tBHQ treatment. (a) Western blotting was performed to detect the expression of p38 MAPKs. (b, c) Expression of VCAM-1 and GATA6. Each group contains the same amount of solvent. Each test was conducted at least 3 times, and a representative blot was shown. nd: none-detective. ^∗^*P* < 0.05 and ^∗∗^*P* < 0.01 indicate statistically significant differences. ns: no significant differences.

We further explored the reasons by which tBHQ decreased GATA6 expression. Previous studies had reported that GATA6 could be regulated by p38 MAPK, which had been implicated in TNF*α*-activated VCAM-1 [49, 50]. Therefore, we detected the effect of tBHQ on p38 MAPK under TNF*α* exposure. Our data showed that tBHQ treatment significantly decreased TNF*α*-activated p38 phosphorylation (Figure 5(a)). SB202190, a special antagonist of p38, markedly inhibited TNF*α*-induced GATA6 upregulation and TNF*α*-activated VCAM-1 expression (Figure 5(b)). However, pretreating vascular endothelial cells with U46619, a special agonist of p38, did not blocked tBHQ-decreased GATA6 and VCAM-1 upregulation (Figure 5(c)). These findings implied that p38 MAPK was independently associated with tBHQ-inhibited GATA6 expression and further VCAM-1 protection.

### 3.6. GATA6 Is Regulated by STAT3 Activation

**Figure 6:**
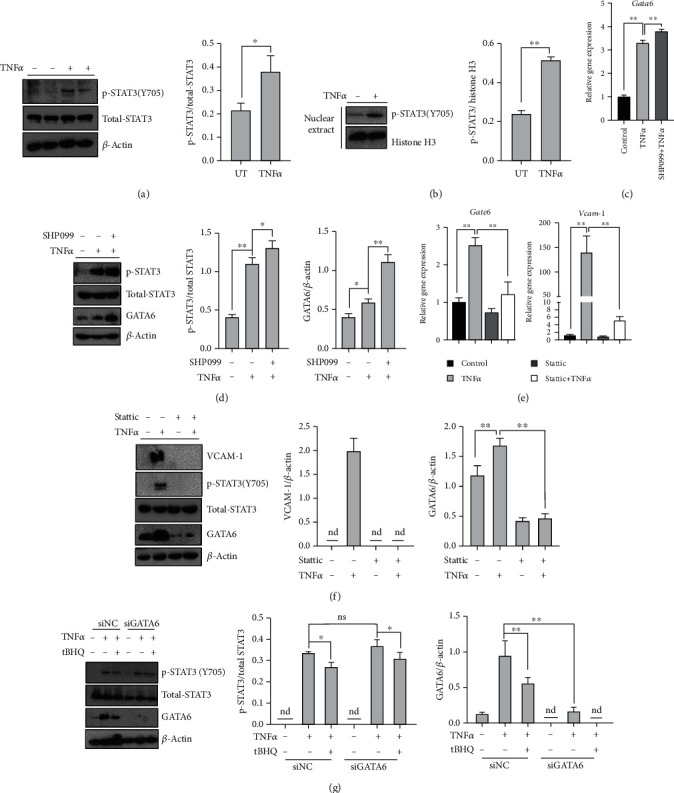
GATA6 is regulated by STAT3 phosphorylation. HAEC cells were treated with TNF*α* (10 ng·mL^−1^, 6 h for mRNA and 16 h for protein detection). tBHQ (100 *μ*mol·L^−1^) was added 1 h before TNF*α* treatment. SHP099 (25 *μ*mol·L^−1^) or Stattic (25 *μ*mol·L^−1^) was added 1 h before TNF*α* treatment. (a, b) Western blotting was performed to detect the expression of total and nuclear phosphorylated STAT3, respectively. (c) The mRNA level of *Gata6*. (d) Western blotting was performed to detect the expression of phosphorylated STAT3 and GATA6. (e) The mRNA levels of *Gata6* and *Vcam-1*. (f) Western blotting was performed to detect the expression of VCAM-1, phosphorylated STAT3, and GATA6. (g) HAEC cells were transfected with siGATA6 or scramble siRNA (siNC) and followed by the indicated treatment. VCAM-1, phosphorylated STAT3, and GATA6 expression were detected by Western blotting. All values are denoted as means ± SD from three or more independent batches of cells. Each group contains the same amount of solvent. nd: none-detective. ^∗^*P* < 0.05 and ^∗∗^*P* < 0.01 indicate statistically significant differences. ns: no significant differences.

In view of GATA6 could be transcriptional regulated by TNF*α*; we subsequently searched the promoter sequence (-2000~0) of GATA6 (NC_000018.10, 22166128-22205837) with Genome Browser (http://www.genome.ucsc.edu/) and predicted several putative binding sites of STAT3 on GATA6 promotor region with JASPAR database (http://jaspar.genereg.net/) (Table 2). TNF*α* exposure significantly induced STAT3 phosphorylation and promoted activated STAT3 to translocate into nucleus (Figures 6(a) and 6(b)). Moreover, maintaining STAT3 activation via its phosphatase inhibitor SHP099 significantly upregulated both mRNA and protein levels of GATA6 in the presence of TNF*α* (Figures 6(c) and 6(d)), whereas inhibiting STAT3 by its chemical antagonist Stattic robustly blocked TNF*α*-stimulated GATA6 upregulation and VCAM-1 activation (Figures 6(e) and 6(f)). Silencing GATA6 by siRNA did not blocked TNF*α*-stimulated STAT3 phosphorylation (Figure 6(g)), which indicated that GATA6 was downstream target of STAT3.

### 3.7. tBHQ Protects against TNF*α*-Induced VCAM-1 Activation via STAT3/GATA6-Dependent Pathway

**Figure 7:**
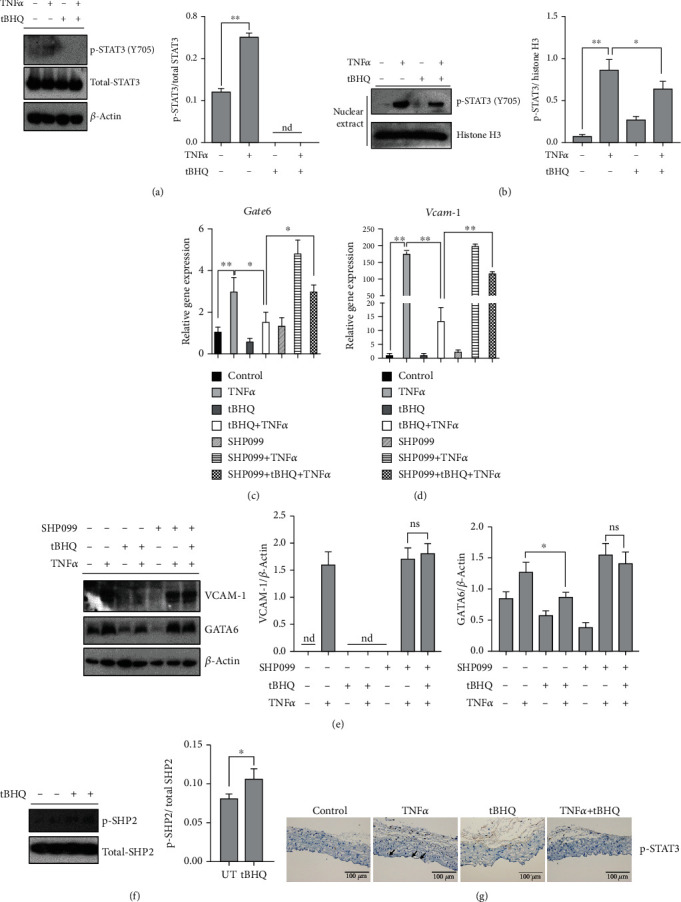
STAT3-regulated GATA6 contributes to tBHQ-protected VCAM-1 activation. HAEC cells were treated with TNF*α* (10 ng·mL^−1^, 6 h for mRNA and 16 h for protein detection). tBHQ (100 *μ*mol·L^−1^) was added 1 h before TNF*α* treatment. SHP099 (25 *μ*mol·L^−1^) was added 1 h before tBHQ treatment. (a) Western blotting was performed to detect the total expression of phosphorylated STAT3. (b) Phosphorylated STAT3 expression in nucleus. (c) The mRNA levels of *Gata6* and *Vcam-1*. (d) *VCAM-1* and *GATA6* expression were detected by Western blotting. (e) Expression for phosphorylated SHP2. (f) Immunohistochemisty for vascular endothelium phosphorylated STAT3 expression (*n* = 5). All values are denoted as means ± SD from three or more independent batches of cells. Each group contains the same amount of solvent. nd: none-detective. ^∗^*P* < 0.05 and ^∗∗^*P* < 0.01 indicate statistically significant differences. ns: no significant differences.

We further investigated whether STAT3-regulated GATA6 pathway conferred to tBHQ-protected VCAM-1 activation. Our data showed that tBHQ treatment significantly reduced TNF*α*-stimulated phosphorylation of STAT3 (Figure 7(a)). TNF*α*-increased intranuclear metastasis of phosphorylated STAT3 was also inhibited by tBHQ intervention (Figure 7(b)). Importantly, maintaining STAT3 activation by SHP099 markedly blocked the protective role of tBHQ against TNF*α*-stimulated GATA6 and VCAM-1 (Figures 7(c) and 7(d)). We also detected the effect of tBHQ exposure on SHP2, a specific phosphatase of STAT3, and observed that tBHQ significantly stimulated SHP2 phosphorylation (Figures 7(e) and 7(f)). More importantly, tBHQ intervention markedly reversed TNF*α* injection-induced increase of phosphorylated STAT3 expression in aortic endothelium (Figure 7(g)).

## 4. Discussion

**Figure 8:**
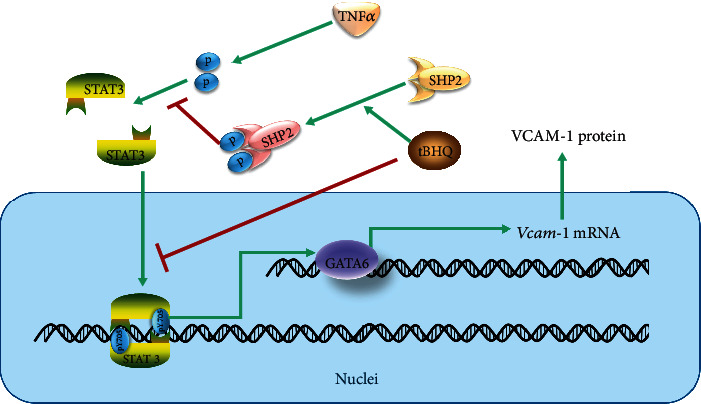
Proposed model for tBHQ-protected VCAM-1 activation induced by TNF*α* in vascular endothelium.

The present study demonstrates for the first time that tBHQ protects TNF*α*-induced VCAM-1 activation in vascular endothelium. The detailed mechanistic investigation reveals that tBHQ-inhibited GATA6 activation induced by TNF*α* via mediating STAT3 phosphorylation contributes to the beneficial role against VCAM-1 activation (Figure 8).

TNF*α*-induced VCAM-1 activation and further dysfunction of vascular endothelial cells play an essential role in the initiation and progression of AS. Therefore, strategy aiming to improve VCAM-1 activation has been well considered as an ideal therapeutic choice for the prevention and treatment of AS. Previous studies revealed that TNF*α* impaired the balance of intracellular redox homeostasis and further induced oxidative stress, which contributed to the activation of VCAM-1 [51, 52]. Ameliorating excessive accumulation of intracellular ROS by antioxidants, including N-acetylcysteine (NAC) [53], clematichinenoside [54], and resveratrol [55], significantly alleviated TNF*α*-induced VCAM-1 activation. tBHQ, a synthetic food antioxidant, is widely found in our diet especially in foods with high lipid content to prevent oils and fats from oxidative deterioration and rancidity. This antioxidant property also empowers tBHQ protective effect in a variety of pathological processes. It has been reported that tBHQ alleviated oxidative stress in methamphetamine-induced chronic pulmonary toxicity [56] and attenuated oxidative stress and inflammation in hypothalamic paraventricular nucleus of high salt-induced hypertension [44]. Through antioxidation, tBHQ also protected different interfering substance-caused dysfunction and even death in various types of cells [30–32, 57]. We therefore hypothesized that tBHQ protects against TNF*α*-induced VCAM-1 activation in vascular endothelium. As expected, our results clearly revealed that tBHQ treatment efficiently attenuated TNF*α*-induced VCAM-1 activation in both cultured human vascular endothelial cells and aortic endothelium and plasma of mice. It is generally conceivable that the activation of Nrf2 by tBHQ confers to its antioxidant property. Additionally, Nrf2 activation has been implicated in the protective effects against different stimuli-induced VCAM-1 activation and endothelial dysfunction [58, 59]. We naturally associated that tBHQ-stimulated Nrf2 activation contributed to the improvement of VCAM-1 upregulation. However, genetically silencing Nrf2 by its special siRNA did not block the protective role of tBHQ, which implying that Nrf2 activation was not strictly involved in this process.

We further explored other potential mechanisms underlining the beneficial effects of tBHQ. Autophagy is a highly evolutionarily conserved catabolic process for the recycling and degradation of certain proteins, lipids, and damaged/aged organelles, playing an essential role in cellular homeostasis [60, 61]. Recently, it has been confirmed that activating autophagy prevented TNF*α*- and oxidized low-density lipoprotein-induced VCAM-1 upregulation in vascular endothelial cells [62]. The induction of autophagic flux helps reducing lipid accumulation, delaying the formation of foam cells and plaque, and removing necrotic cells in different stages of AS [63]. Previously, we reported that tBHQ is a strong autophagy inducer in hepatocytes [45]. Therefore, we proposed that autophagy induction might exist in tBHQ-exposed vascular endothelial cells and be involved in its protective role. In the present study, we observed that tBHQ indeed activated autophagic flux in vascular endothelial cells. Activating autophagy by rapamycin also prevented TNF*α*-induced VCAM-1 upregulation. However, blocking autophagy failed to counteract the protective effects of tBHQ to restore VCAM-1 induced by TNF*α*, indicating that other unknown mechanisms might also be involved.

Vascular endothelial cells are the main resource for VCAM-1 production. In the resting state, the intracellular content of VCAM-1 is extremely low and hard to be detected by Western blotting. However, it could be rapidly transcriptional-activated by proatherosclerotic cytokines (including TNF*α*) in both human endothelial cells and experimental animals [10, 12, 64]. Several nuclear transcriptional factors of VCAM-1, including NF-*κ*B (p65), GATA6, SP-1, c-Fos, and c-Jun, have been identified in the past decades. Accumulated evidence has confirmed that genetically silencing or pharmaceutical inhibiting the transcription factors mentioned above could effectively alleviate different stimulus-caused VCAM-1 activation [5, 15–19]. In the present study, we observed that TNF*α* treatment only increased the nuclear contents of NF*κ*B (p65) and GATA6, but not SP-1, c-Fos, and c-Jun. The inconsistent results of TNF*α*-induced SP-1, c-Fos, and c-Jun from previous studies might be attributed to different experimental settings. Importantly, we found that only TNF*α*-induced GATA6 entering into the nucleus could be reversed by tBHQ treatment, implying that GATA6 might be involved in the beneficial role of tBHQ. Mammalian GATA6 (also been known as transcription factor GATA6), containing conserved tandem zinc finger structure, is indispensable for the development and tissue-specific gene regulation via its DNA-binding ability [65]. In the presence of TNF*α*, GATA6 is stimulated to translocate into the nucleus and further binds with the promoter region of VCAM-1 gene [25]. The exact mechanism behind TNF*α*-induced GATA6 binding with VCAM-1 promotor is still obscure. Several partners of GATA6 including transcriptional coactivator p300, NFAtc1, and thyroid transcription factor-1 (also termed as Nkx2.1) had been confirmed to interact with GATA6 and regulate gene transcription in other tissues or cultured cells [66–68]. Although whether the coactivators mentioned above were involved in TNF*α*-induced GATA6 and VCAM-1 was not explored in this study, we interestingly observed that TNF*α* exposure significantly upregulated both mRNA and protein levels of GATA6. In support of our findings, a previous study also reported that TNF*α* transcriptionally stimulated GATA6 in human umbilical vein endothelial cells [25]. Besides, tBHQ intervention significantly reversed TNF*α*-induced total expression of GATA6 in both cultured human vascular endothelial cells and aortic endothelium. Our result also revealed that genetically silencing GATA6 by its special siRNA markedly alleviated TNF*α*-activated VCAM-1, indicating that the intracellular abundance of GATA6 was critical for VCAM-1 upregulation. However, the transcriptional regulation of TNF*α* on GATA6 is still unclear so far. A recent study revealed that p38 MAPK pathway participated in the regulation of GATA6 expression in mouse preimplantation embryos [49]. Considering the known evidence that p38 MAPK was involved in TNF*α*-activated VCAM-1, we therefore proposed that a p38 MAPK-regulated GATA6 pathway existed in the regulation of TNF*α*-activated VCAM-1 and also the protective role of tBHQ. In line with our vision, tBHQ treatment obviously reversed TNF*α*-stimulated phosphorylation of p38 MAPK, and inhibiting p38 MAPK by its special antagonist markedly reversed TNF*α*-activated expression of GATA6 and further VCAM-1. Out of our expectation, activating p38 MAPK by its chemical agonist did not blocked tBHQ-decreased GATA6 expression, which excluded the participation of p38 MAPK. Additionally, it was known that GATA6 could be phosphorylated at its serine sites at Ser^37^, Ser^120^, Ser^264^, and Ser^265^ by JNK MAPK, GSK3, and ERK MAPK phosphorylation, respectively [69–72]. Although TNF*α* could stimulate the phosphorylation of both JNK MAPK and GSK3 in various types of cells [73–75], their phosphorylation led to GATA6 degradation but not activation, which was contradictory to TNF*α*-stimulated GATA6 upregulation. We further detected the effect of tBHQ on TNF*α*-phosphorylated ERK1/2 and observed that tBHQ treatment significantly reversed TNF*α*-stimulated ERK1/2 phosphorylation (Figure S5(a)). However, inhibiting ERK1/2 via its special antagonist did not block TNF*α*-stimulated VCAM-1 expression (Figure S5(b)), which excluded the involvement of ERK1/2-regulated GATA6 pathway in the beneficial role of tBHQ.

In the light of limited study has addressed the definite nuclear factors for transcriptional regulation of GATA6 in the presence of TNF*α*. We further predicted putative binding sites in the promotor sequence of GATA6 via JASPAR database (http://jaspar.genereg.net/). We found that there exist multiple STAT3-binding sites within the promoter of GATA6, implying that STAT3 is a potential nuclear factor of GATA6. STAT3 was firstly found in 1988 as protein that bind to interferon- (IFN-) stimulated response elements of DNA sequences to stimulate the transcription of type I IFNs. Upon phosphorylation, STAT3 dimerized and entered to the nucleus to initiate transcription of myriad target genes, transferring signals from cell membrane receptors to the nucleus [76]. In this study, we observed that TNF*α* treatment significantly activated STAT3 phosphorylation and promoted translocation of activated-STAT3 into nucleus. Importantly, increasing the stability of phosphorylated STAT3 via inhibiting its special dephosphorylase markedly prompted both transcriptional and protein upregulation of GATA6 by TNF*α*. Meanwhile, inhibiting STAT3 phosphorylation via its special antagonist significantly abolished TNF*α*-induced activation of GATA6 and VCAM-1. The above evidences clearly revealed that the activity of STAT3 was involved in TNF*α*-regulated GATA6 expression. Although previous studies had also addressed the involvement of STAT3 in TNF*α*-induced VCAM-1 [24], limited study had proclaimed how STAT3 regulated VCAM-1. In this study, we demonstrate for the first time that GATA6 is essential for STAT3-regulated VCAM-1, evidenced by our observation that genetically silencing GATA6 robustly alleviated TNF*α*-induced VCAM-1 but not STAT3 phosphorylation. More importantly, we confirmed that tBHQ treatment significantly abolished TNF*α*-phosphorylated STAT3 and further translocation into the nucleus. Maintaining STAT3 phosphorylation effectively limited the protective role of tBHQ on GATA6 and VCAM-1 expression. These data strongly supported that tBHQ protected TNF*α*-activated VCAM-1 via a novel STAT3-mediated GATA6 pathway.

In summary, we provided strong evidence for the first time that tBHQ protected against TNF*α*-induced VCAM-1 activation in mice aortic endothelium and cultured human vascular endothelial cells. Our study also identified a novel STAT3-regulated GATA6 pathway contributing to the beneficial role of tBHQ, which was independent of Nrf2 and autophagy activation. However, further studies are still needed to explore whether tBHQ intervention protects against atherosclerotic plaque formation via alleviating VCAM-1 activation in AS animal model. Our findings suggest that tBHQ may be a potential candidate for the prevention of inflammatory factor-caused vascular endothelial dysfunctions.

## Figures and Tables

**Table 1:** List of primers.

Gene	Forward primer (5′-3′)	Reverse primer (5′-3′)
*Vcam-1*	TTCCCTAGAGATCCAGAAATCGAG	CTTGCAGCTTACAGTGACAGAGC
*Nrf2*	TTCCCGGTCACATCGAGAG	TCCTGTTGCATACCGTCTAAATC
*Gata6*	GTGCCAACTGTCACACCACA	GAGTCCACAAGCATTGCACAC
*ACTB*	CATGTACGTTGCTATCCAGGC	CTCCTTAATGTCACGCACGAT

**Table 2:** Putative binding sites of STAT3 on promoter of GATA6.

Matrix	Score	Predicted sequence
MA0144.2	11.7378	ctgccggaaaa -1728~-1739 bp
	7.37868	gtttcgggaac -1053~-1064 bp
	6.75067	cttacagaaag -588~-599 bp
	6.67458	tttcaggtaag -870~-881 bp
	6.41008	ctcctagaaag -420~-431 bp
	6.105	ctaccgagaag -93~-104 bp
	2.82235	tttgtgagaat -511~-522 bp
	2.70843	aattcaggaag -173~-184 bp
	2.12988	tctcctagaaa -421~-432 bp
	1.57891	cagaatggaaa -962~-973 bp
	1.09258	tttccagagat -295~-306 bp
	0.723657	ctgctggtgaa -1771~-1782 bp
	0.714859	ttggcgagaat -162~-173 bp

## Data Availability

Readers can access the data supporting the conclusions of the study from the corresponding author Songtao Li.

## Supplementary Materials

Supplementary Materials The supplementary material contains the following figures in one file. Figure S1: negative controls for the immunohistochemisty. Figure S2: tBHQ protects TNF*α*-induced VCAM-1 activation in vascular endothelium. Figure S3: autophagy induction is negatively associated with tBHQ-prevented VCAM-1 activation. Figure S4: tBHQ inhibits TNF*α*-activated GATA6 in vascular endothelial cells. Figure S5: ERK1/2-regulated GATA6 contributes to the beneficial role of tBHQ. Figure S6: (a) HAEC cells were transfected with si Nrf2 or scramble siRNA (si NC); 24 h later, cell viability was detected by CCK-8 method. (b) Nrf2 protein expression under the treatment of si NC or si Nrf2. (c) LC3 puncta were detected by confocal microscope after 16 h tBHQ treatment. Scale bar, 5 *μ*m.
