# Supplementary material for: A Novel STAT3-Mediated GATA6 Pathway Contributes to tert-Butylhydroquinone- (tBHQ-) Protected TNFα-Activated Vascular Cell Adhesion Molecule 1 (VCAM-1) in Vascular Endothelium
Source: Oxid Med Cell Longev. 2020 Nov 14;2020:6584059. doi: 10.1155/2020/6584059 (PMC7683157; doi:10.1155/2020/6584059)
Supplement: Supplementary Materials — The supplementary material contains the following figures in one file. Figure S1: negative controls for the immunohistochemisty. Figure S2: tBHQ protects TNFα-induced VCAM-1 activation in vascular endothelium. Figure S3: autophagy induction is negatively associated with tBHQ-prevented VCAM-1 activation. Figure S4: tBHQ inhibits TNFα-activated GATA6 in vascular endothelial cells. Figure S5: ERK1/2-regulated GATA6 contributes to the beneficial role of tBHQ. Figure S6: (a) HAEC cells were transfected with si Nrf2 or scramble siRNA (si NC); 24 h later, cell viability was detected by CCK-8 method. (b) Nrf2 protein expression under the treatment of si NC or si Nrf2. (c) LC3 puncta were detected by confocal microscope after 16 h tBHQ treatment. Scale bar, 5 μm. [file 6584059.f1.docx]

**Supplementary Material**


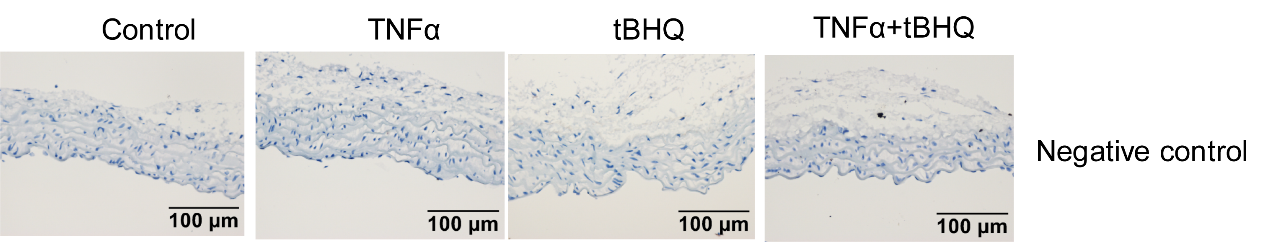


Figure S1. Negative controls for the immunohistochemisty.


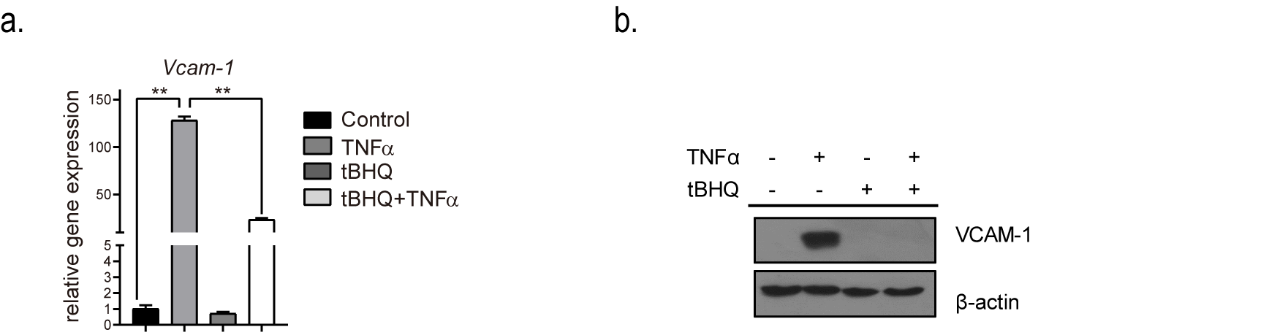


Figure S2. tBHQ protects TNFα-induced VCAM-1 activation in vascular endothelium. (a) EA.hy926 cells were pretreated with tBHQ (100 μmol·L^-1^) for 1 h and then followed by TNFα (10 ng·mL^-1^) stimulation for 6 h. The mRNA level of *Vcam-1* was detected. (b) EA.hy926 cells were treated with TNFα (10 ng·mL^-1^) for 16 h. tBHQ was added 1 h before TNFα treatment. Protein expression of VCAM-1 was detected. All values are denoted as means ± SD from three or more independent batches of cells. Each group contains the same amount of solvent. ** (*P* < 0.01) indicates statistically significant differences.


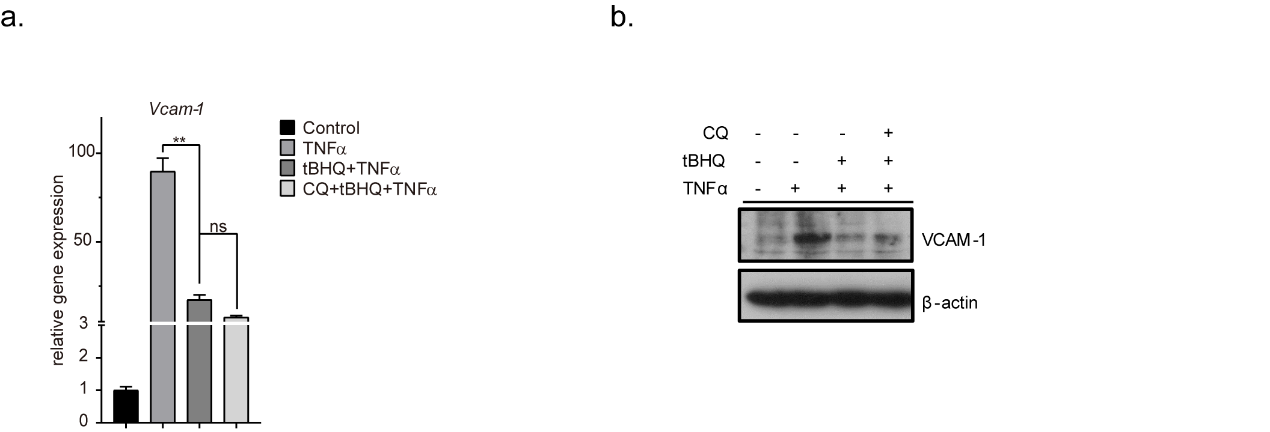


Figure S3. Autophagy induction is negatively associated with tBHQ-prevented VCAM-1 activation. EA.hy926 cells were treated with TNFα (10 ng·mL^-1^, 6 h for mRNA and 16 h for protein detection). tBHQ (100 μmol·L^-1^) was added 1 h before TNFα treatment. CQ (20 μM) or Baf A1 (1 μmol·L^-1^) were added 1 h before tBHQ intervention. (A) mRNA of *Vcam-1*. (B) VCAM-1 expression was detected by Western-blotting. All values are denoted as means ± SD from three or more independent batches of cells. Each group contains the same amount of solvent. ** (*P* < 0.01) indicates statistically significant differences. NS, no significant differences.


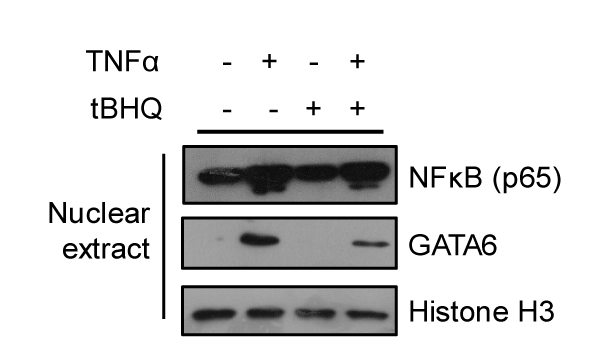


Figure S4. tBHQ inhibits TNFα-activated GATA6 in vascular endothelial cells. EA.hy926 cells were treated with TNFα (10 ng·mL^-1^) for 16 h. tBHQ (100 μmol·L^-1^) was added 1 h before TNFα treatment. Nuclear protein was exacted after the indicated treatment. Western-blotting was performed to detect the expression of NFκB (p65), and GATA6.


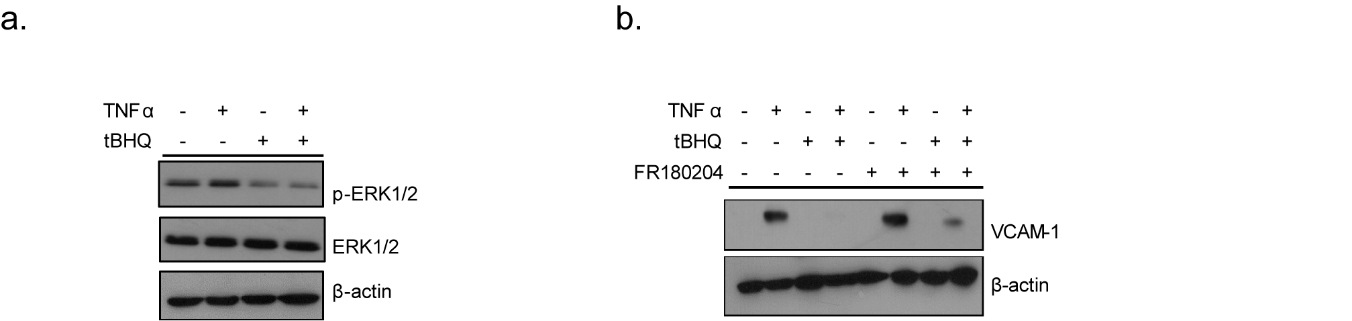


Figure S5. ERK1/2-regulated GATA6 contributes to the beneficial role of tBHQ. HAEC cells were treated with TNFα (10 ng·mL^-1^) for 16 h. tBHQ (100 μmol·L^-1^) was added 1 h before TNFα treatment. FR180204 (10 μmol·L^-1^) was added 1 h before tBHQ treatment. (A) Western-blotting was performed to detect the expression of ERK1/2 MAPK. (B) Expression of VCAM-1 and GATA6. Each group contains the same amount of solvent.


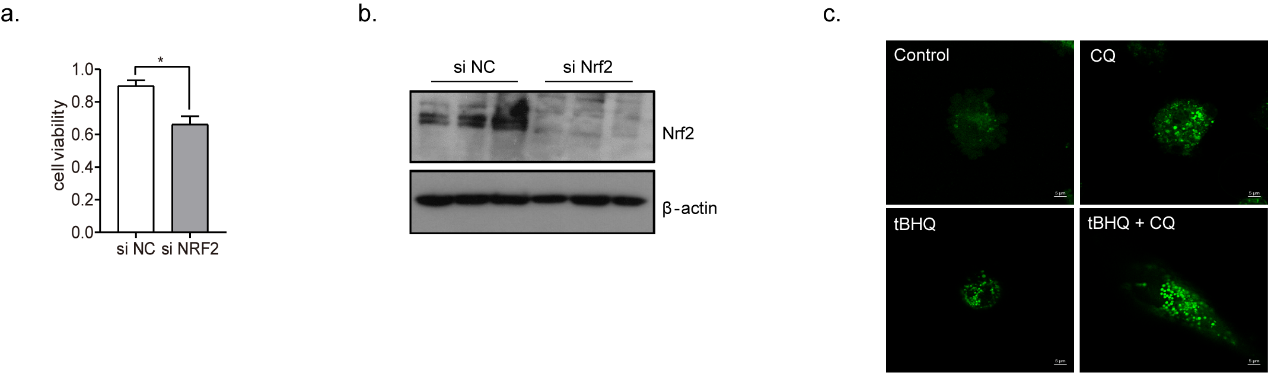


Figure S6. (a) HAEC cells were transfected with si Nrf2 or scramble siRNA (si NC), 24 h later, cell viability was detected by CCK-8 method. (b) Nrf2 protein expression under the treatment of si NC or si Nrf2. (c) LC3 puncta were detected by confocal microscope after 16 h tBHQ treatment. Scale bar, 5 μm.
